# Polymeric Lipid Hybrid Nanoparticles (PLNs) as Emerging Drug Delivery Platform—A Comprehensive Review of Their Properties, Preparation Methods, and Therapeutic Applications

**DOI:** 10.3390/pharmaceutics13081291

**Published:** 2021-08-18

**Authors:** Durgaramani Sivadasan, Muhammad Hadi Sultan, Osama Madkhali, Yosif Almoshari, Neelaveni Thangavel

**Affiliations:** 1 Department of Pharmaceutics, College of Pharmacy, Jazan University, Jazan 114, Saudi Arabia; mhsultan@jazanu.edu.sa (M.H.S.); omadkhali@jazanu.edu.sa (O.M.); pooja2004@gmail.com (Y.A.); 2 Department of Pharmaceutical Chemistry, Jazan University, Jazan 114, Saudi Arabia; venipsgcop2004@yahoo.co.in

**Keywords:** polymeric lipid nanoparticles, high-pressure homogenization, nanomedicine, quality by design approach, drug delivery mechanisms

## Abstract

Polymeric lipid hybrid nanoparticles (PLNs) are core–shell nanoparticles made up of a polymeric kernel and lipid/lipid–PEG shells that have the physical stability and biocompatibility of both polymeric nanoparticles and liposomes. PLNs have emerged as a highly potent and promising nanocarrier for a variety of biomedical uses, including drug delivery and biomedical imaging, owing to recent developments in nanomedicine. In contrast with other forms of drug delivery systems, PLNs have been regarded as seamless and stable because they are simple to prepare and exhibit excellent stability. Natural, semi-synthetic, and synthetic polymers have been used to make these nanocarriers. Due to their small scale, PLNs can be used in a number of applications, including anticancer therapy, gene delivery, vaccine delivery, and bioimaging. These nanoparticles are also self-assembled in a reproducible and predictable manner using a single or two-step nanoprecipitation process, making them significantly scalable. All of these positive attributes therefore make PLNs an attractive nanocarrier to study. This review delves into the fundamentals and applications of PLNs as well as their formulation parameters, several drug delivery strategies, and recent advancements in clinical trials, giving a comprehensive insight into the pharmacokinetic and biopharmaceutical aspects of these hybrid nanoparticles.

## 1. Introduction of Lipid-Based Drug Delivery Systems

Nanotechnology is a plausible medical technology with the ability to significantly affect the distribution of a wide range of therapeutics, including genomes, peptides, small molecular RNA therapeutics, and medical imaging agents, as well as to enhance the pharmacokinetics and therapeutic index of many medicines systemically [1]. After they have been systemically integrated, these payloads are covalently embedded on the surface of the nanocarriers or encapsulated within, and their release is regulated by a variety of factors, such as the matrix composition, microenvironment pH, and surrounding temperature [2,3]. A small number of core parameters, such as the average nanometric distance, surface potential, homogeneity, and drug loading, are primarily responsible for the intrinsic potential of nanoparticles for therapeutic cargo distribution [4]. The reticuloendothelial system can be evaded by surface-coated immuno-inert nanoparticles, thereby improving the bioavailability of encapsulated drugs [5]. Some of the possible benefits of nanocarriers include the enhancement of a drug’s pharmacodynamic and pharmacokinetic properties holistically without changing its molecular structure, improved and effectual molecular targeting of the cells and tissues, the ability to overcome several inherent impediments in vivo, the targeted and non-targeted delivery of drugs to the target, e.g., nucleus, cytosol, etc., and better drug therapy [6,7].

Recent advancements in synthetic chemistry and pharmaceutical research have concentrated on novel drug delivery mechanisms that use innovative systems to target and alter the delivery time of drugs as well as boosting the in vivo solubility and bioavailability of poorly soluble drugs. Combinatorial chemistry advancements have resulted in a huge upsurge in sparingly soluble drugs, with 40–70% of the modern active pharmacological substances having truncated aqueous solubility [8]. Several preparation methods are currently used to address the formulation problems faced by drugs categorized into classes II and IV according to the Biopharmaceutical Classification System (BCS). This can be achieved in one of two ways: using an appropriate solvent and then encapsulating it into capsules, or using water-soluble polymers to form a powder [8,9]. Such methods are likely to overcome the difficulty of the drug’s dissolution in the aqueous environments of the GI tract to some extent; nevertheless, significant issues such as drug precipitation during GIT dispersion or drug crystallization in the matrix of the polymer remain unsettled. The use of lipid-based drug delivery systems has successfully resolved these issues [8,10]. The increasing production of lipid excipients with unique properties calls for increased and versatile applicability in the context of altering their drug release profiles and the bioavailability of the sparingly soluble drugs [10].

Lipid-based preparations can be utilized for their impact on active ingredient absorption via a variety of pathways. They have the ability to alter the intestinal environment, trigger the lymphatic transportation of the active ingredients, and communicate with enterocyte-based transport [11]. Drug processing is increased significantly when the drug is submerged in the lipid-formed matrix of the drug delivery system (LDDS) [12,13,14,15,16]. Fatty acids, glycerides, triglycerides, non-ionic and ionic surfactants, and other lipid-based excipients are considered to be permeability enhancers, possibly due to the elevated fluidity of the membrane [14]. The association of LDDS with efflux transporters also improves the permeability.

**Figure 1:**
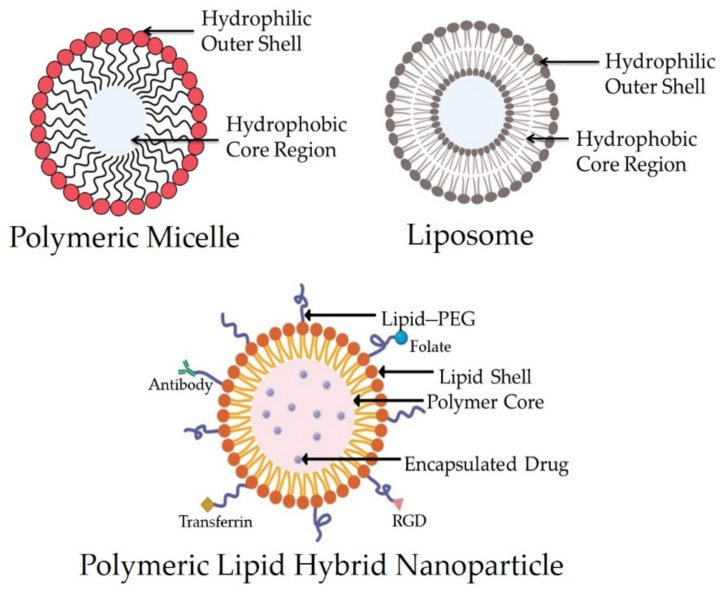
Structure of a polymeric micelle, liposome, and polymeric lipid hybrid nanoparticle (PLN) displaying the organization of hydrophobic and hydrophilic cores. Abbreviation: RGD—arginylglycyl aspartic acid.

**Figure 2:**
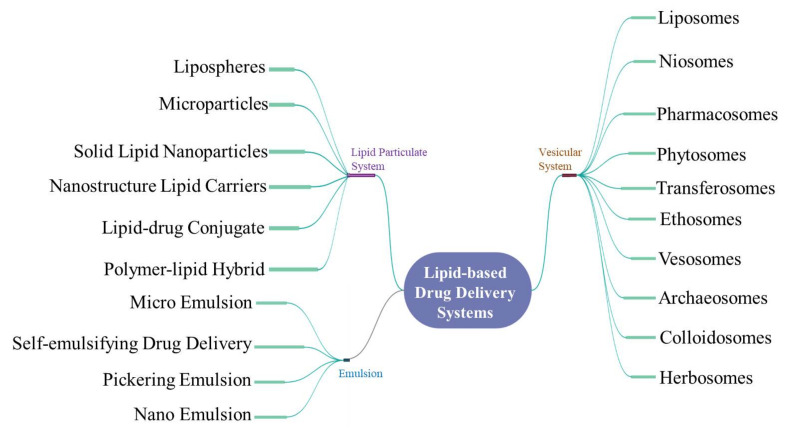
Mind map depicting the various lipid-based drug delivery systems.

Liposomes are the vesicles made by dispersing natural or synthetic amphipathic lipids in water to form one or more bilayers (Figure 1). Since their invention, they have mostly been used for targeted delivery due to their increased biocompatibility and efficacy and safety profiles (Figure 2). Polyethylene glycol (PEG) may be attached to their surface, which extends the circulating half-life [17,18]. The Food and Drug Administration (FDA) licensed Doxil and Myocet, two of the popular doxorubicin liposomes, in 1995 and 1999, respectively, thereby paving the way for many others [19]. Only a few liposomal drugs have been approved for therapeutic use: AmBisome (amphotericin B), DaunoXome (daunorubicin), DepoCyt (cytarabine), DepoDur (morphine), and Visudyne (verteporfin) are some of the licensed liposomal drugs for routine clinical usage [20]. Despite FDA approval, none of the FDA-approved liposomal drugs have demonstrated any significant improvement in overall survival (OS) rates as compared to the parent drug until recently [21].

On the other hand, polymeric nanoparticles are made through the self-assemblage of biodegradable amphiphilic copolymer blocks with a variable hydrophobicity that can be injected into the body. Polymeric NPs have a core–shell structure that helps them to encapsulate hydrophobic materials and increase the drug release and circulation time. Their surfaces may be ligand-decorated for selective drug delivery. Genexol-PM, for example, is a paclitaxel (PCX) and poly(D,L-lactide-co-glycolide)-b-poly (ethylene glycol) methyl ether (PLGA-mPEG)-based NP formulation that is accepted for the treatment of breast carcinoma in Europe and Korea [22].

A new generation of delivery systems, named polymeric lipid hybrid nanoparticles (PLNs) (Figure 1), was developed to take advantage of the special properties of polymeric nanoparticles and liposomes that contributed to their early therapeutic effectiveness, while also addressing disadvantages such as their structural disintegration, shortened circulation period, and material leakage [23]. This hybrid system can be a sturdy drug delivery mechanism with excellent drug release kinetics, high encapsulation efficiency, and distinct drug uptake as well as appropriate tissue and molecular targets [24]. In this review, we highlight some of the newest PLN developments, including innovative processing approaches and drug delivery strategies in therapy.

## 2. The Polymeric Lipid Hybrid Nanoparticles (PLNs)

Polymeric nanoparticles have demonstrated considerable promise in targeted drug delivery for the treatment of a plethora of diseases. The term “nanoparticle” encompasses the characteristics of both nanocapsules and nanospheres, which vary morphologically. Nanocapsules (reservoir system), have a fatty kernel in which the drug is generally dispersed and a polymeric shell that regulates the profile of the drug’s release from the core. The drug can be sustained within or adsorbed onto the surface of nanospheres (matrix system), which are made up of a continuous polymeric network [25].

Apart from the existing advantages, polymeric NPs demonstrate the distinct drawbacks of toxic decay and toxic monomer accumulation, as well as a toxic degradation mechanism that hinders their utility [26]. The lipid NPs also exhibit poor drug-loading capacities, low membrane retention properties, physical state instability of lipids, and diminished cell membrane fluidity, all of which contribute to lipid NPs’ loss of stability (drug expulsion, gelation, etc.) during storage and administration.

To overcome the limitations of both lipid NPs and polymeric NPs, polymeric lipid hybrid nanoparticles (PLNs) have emerged. The word “hybrid” is used as the NPs have the properties of both polymeric and lipid particles [27]. The polymer regulates the drug release, while the utility of the lipid is to improve the permeation of drugs across the membrane and loading. Polymeric lipid hybrid nanoparticles (PLNs) have the ability to improve the biocompatibility and physical stability of drugs, paving the way for their promising use in robust drug delivery.

### 2.1. Structure and Components of PLNs

The architecture of PLNs consists of a monolithic matrix PLN and a core–shell PLN [28]. The first type contains a drug–polymer complex and an siRNA (small interfering RNA)–polymer complex, both of which are uniformly distributed in the rigid solid lipid matrix, with the polymer–siRNA complex having targeting moieties on the surface. The latter type contains two kinds of PLN: PLN with drug in the core and PLN with siRNA attached at the core–shell interface. The solid matrix core, phospholipid, and PEGylated phospholipid form the membrane, which traps the polymers and drugs within. During the one-step method preparation, a phospholipid monolayer is formed. From the formed lipid films or liposomes, a phospholipid bilayer can be produced by the thin-film dispersion method [29].

**Figure 3:**
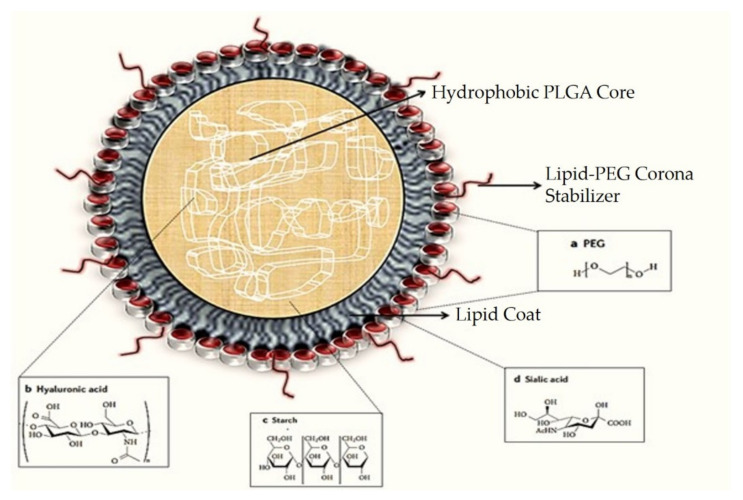
A typical polymeric lipid hybrid nanoparticle (PLN) is depicted in the above representation. These PLNs are made up of a PLGA (polylactic-co-glycolic acid) core, which is hydrophobic in nature, hydrophilic polymer coatings, and a lipid monolayer at the interface between the two.

PLNs are made up of three elements that combine the properties of polymeric nanoparticles and liposomes (Figure 3). They consist of a biocompatible hydrophobic polymeric kernel encasing the therapeutic compounds, a phospholipid monolayer, and an outer PEG layer that serves as a furtive covering and delays the PLNs’ circulation period in vivo while also rendering steric stabilization. During the preparation of PLNs, the inner lipid layer also serves as a molecular fence, preventing the encapsulated fluid from escaping. Furthermore, the inner lipid layer delays the polymer degradation rate of the PLN product by preventing inward water diffusion, resulting in sustained content release kinetics.

The PLNs have strong structural integrity, storage stability, and controlled release capability due to their core–shell architecture. Their strong biocompatibility and bioavailability are also due to the lipid and outer PEGylated layers [30]. Due to their outstanding qualities, PLNs have proven to be a reliable drug delivery mechanism. On that note, utilizing methoxyl groups to modify the end group of the PEG chains of the lipid–PEG reduces the immune system’s activation level against PLNs, resulting in lower immunogenicity. These factors aptly demonstrate the efficacy and versatility of PLNs as a drug delivery system [31].

### 2.2. Advantages and Classification of PLNs

The seamless integration of polymers and lipids in this kind of system brings various advantages, including the following:

1. Enhanced stability and better biocompatibility owing to the lipid–PEG shell;
2. Controlled or regulated delivery because of the polymeric core.

**Figure 4:**
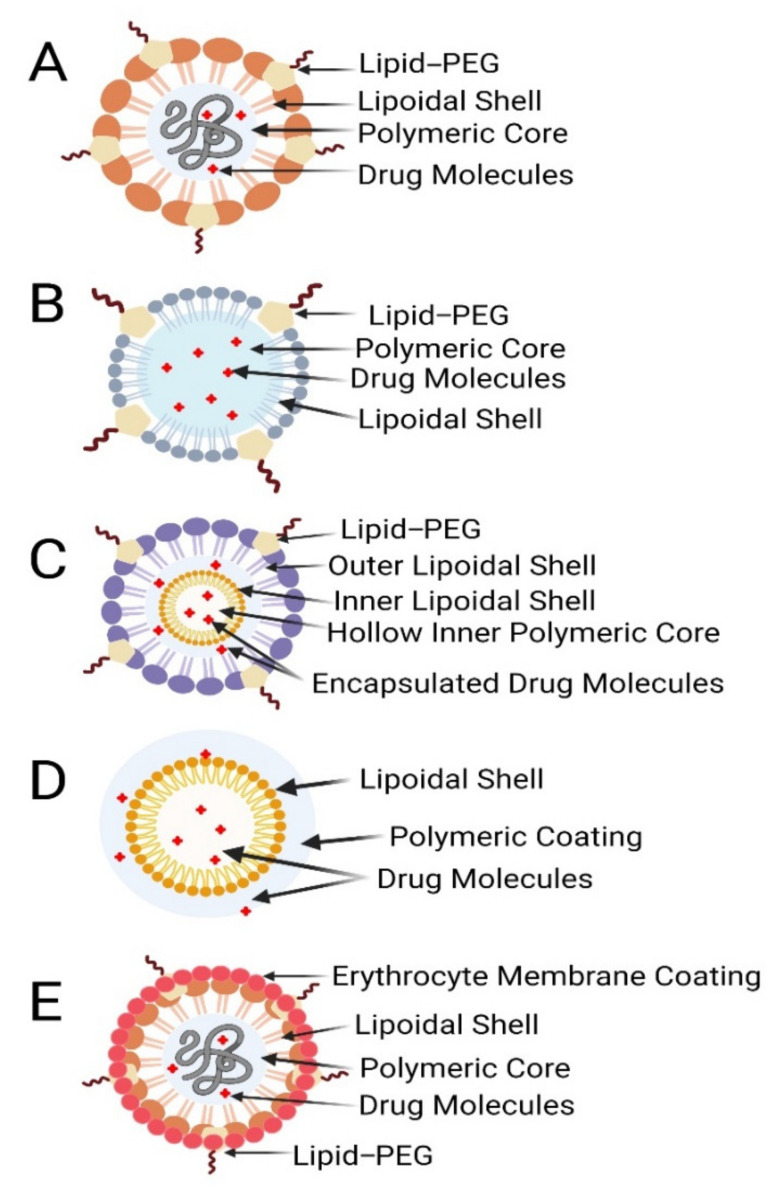
Different types of polymer–lipid hybrid nanoparticles. (**A**) Polymer core, lipid shell. (**B**) Polymer-caged nanobins. (**C**) Core shell-type hollow lipid–polymer–lipid hybrid nanoparticles. (**D**) Monolithic PLNs. (**E**) Erythrocyte membrane-coated PLNs.

All of these key tenets have now been integrated into a single system known as a polymeric lipid hybrid nanoparticle [30]. There are several combinations of polymers and lipids that can be made. The limitations of individual structures are reported to be mitigated in any hybrid framework. Polymers are known to play a critical role in sustained drug release in hybrid systems. The polymers’ architectural stability further increases the system’s durability in general. On the other hand, the importance of lipids cannot be overstated: they have many benefits as a result of their biomimetic existence, and they improve the carrier system’s drug-loading ability. In comparison to all of the particles alone, the hybrid particles have better and longer in vivo efficacy [23]. Since all lipids and polymers are fused in a single form, these hybrid structures are potentially very beneficial delivery systems [32]. The following are some of the different types of PLN structures (Figure 4), which are classified based on how the lipids and polymers are organized (Table 1):

- Polymer core–lipid shell hybrid NPs;
- Lipid-bilayer-coated NPs;
- Polymer-caged nanobins;
- Monolithic PLN/mixed polymer–lipid hybrid NPs;
- Hollow-core NP/lipid–polymer–lipid NPs.

Polymer core–lipid shell hybrid NPs: The most significant in terms of drug delivery and the most studied of all of the above are the hybrid polymer core–lipid shell fragment nanoparticles [32]. These nanoparticles have a polymeric core and a lipid shell. The first applications to use such systems were biomedical and biotechnological devices [33]. To make hybrid nanoparticles, liposomes are mixed with polymeric NPs to create a composite assembly with a polymeric core and a lipid coating. One or two layers of lipids surround the polymer backbone, with water or buffer filling in the holes. Shells can be made from both cationic and anionic phospholipids. Electrostatic encounters of oppositely charged moieties can be aided by the ionic character [32]. Zhang et al. discovered that the hybrids’ characteristic structural properties are the result of three main layers: (a) a biodegradable hydrophobic polymer PLGA (polylactic-co-glycolic acid), (b) a lipid–PEG conjugate shell on the exterior with the ability to integrate hydrophilic drugs, and (c) a third layer between the inner and outer shell that is composed of lipids that function as a barrier on both sides. As a result, this prevents the inner polymeric material from leaking or water from penetrating from the outside. Such a framework ensures a consistently robust formulation of maximum structural integrity [23]. Hydrophobic drugs can be easily inserted into this type of PLN, but it is hard to incorporate water-soluble ionic drugs. A stable complex formation of an ionic drug with the counter ion polymer may improve the loading of ionic drugs to PLNs. The system’s integrity is further enhanced by the outer lipid shell. Dong et al. described PLNs as SLNs (solid lipid nanoparticles) that are modified to hold a positively charged hydrophilic drug within an anionic polymer [34]. Salidroside (Sal), a potent anti-tumor compound that is water soluble, was entrapped in a polymeric core–lipid shell (PLGA–PEG–PLGA), leading to improved entrapment capacity, increased tumor cell uptake, and smaller particle size [35]. Another instance is the encapsulation of Enoxaparin in alginate-coated chitosan nanoparticles, where the cationic polymer chitosan serves as a charge stabilizer [36].

Lipid-bilayer-coated nanoparticles: Other approaches have been investigated in circumstances in which the presence of PEG alone is insufficient to provide in vivo particle stabilization. Red blood cell (RBC) membrane-derived particles are lipid-bilayer-coated NPs. The heat treatment of RBCs is often used to make those NPs. The polymeric nanoparticles are encased in the erythrocyte membrane. The drug is targeted within the polymeric NPs; this provides the particles with biological surface strength, ensuring maximum in vivo stability [37]. RBCs are compacted, and vesicles derived from the erythrocyte membrane are formed. The drug is encased in vesicles that imitate the complex surface chemistry of RBCs [38]. The drug is released from the lipid bilayer in a prolonged manner. Moreover, because of the dense lipid barrier, the RBC may exhibit a slower release. Since the RBCs from various blood types contain varied forms of antigens, cross-matching during blood transfusion can be difficult [39].

Polymer-caged nanobins: To obtain the desired properties, such as enhanced drug release, improved drug stability, and reduced drug leakage, these particles have an additional coating of polymers on the outside. Outer layers of polyacrylic acid, for example, can have additional surface features. It would be possible to connect various types of linkers to these using carboxylic groups, resulting in pH-sensitive actions [40]. Liposomes with polymers are liposomes with surface properties that are modified to achieve optimal characteristics. The first step in the preparation process is to create liposomes. These are then supplemented by the application of polymeric coats to their surfaces, such as cholesterol-functionalized poly acrylic acid. Carboxylate groups are added to the liposomal surfaces in this manner, giving them further functionality [40]. The features of the release of drugs can be modified. Another approach protecting the environment of the liposome is to use a pH-responsive cross-linked polymer. The polymer coating has enormous benefits that include the protection of liposomes, promotion of durability, reduction in drug degradation due to leakage, and an increase in drug release where it is required [41].

Monolithic LPNPs/mixed lipid–polymer NPs: These are particles that have lipids scattered in a polymer matrix. This is an innovation that can be compared to a colloidal carrier for drug distribution [42]. They are made up of a mixture of copolymers (which are amphiphilic in nature) and lipids. Liposomes are made up of the same components as cells. Phospholipids are the crucial components of their framework and can form a vesicular structure [43]. Phospholipids, on the other hand, are not stable enough to enable structural alteration by PEGylation as PEG–lipids at higher PEG densities could form micelles.

Hollow-core NPs/lipid–polymer–lipid NPs: These are concentric structures of the A–B–A type, with an innermost hollow layer of lipid. The polymer makes up the following middle layer. The outermost layer is made up of lipid–polyethylene glycol (PEG) [44]. The double emulsification solvent evaporation method may be used to make these lipid–polymer–lipid NPs. They have both polymer lipoplexes and poly lactic-co-glycolic acid (PLGA) nanoparticle properties [23]. It is not taken up by macrophages because of the outer lipid–PEG-conjugated substrate, which boosts these particles’ in vivo stability. Such devices improve the stability and protection of the inner polymer sheet [44]. In comparison to the polymeric core, the inner cationic lipid layer contains sufficient anionic drugs; at the same time, this mechanism can encapsulate two medications. For MDR cancer, for example, loading siRNA and a small drug moiety into a hydrophobic kernel can be carried out to achieve co-delivery [45].

**Table 1:** Comparison of polymeric lipid hybrid nanoparticles [46].

Type of PLNs	Advantages	Disadvantages
Polymer core–lipid shell type	Improved encapsulation efficiency over liposomes. Optimization of core–shell layer will promote sustained release profile of drugs. Lipophilic and hydrophilic drugs can be easily entrapped	Poor drug-loading and entrapment efficiency
Core–shell-type hollow lipid–polymer nanoparticles	Delivery of si-RNA and mRNA by reducing its susceptibility to serum nucleases and phagocytic uptake	Toxicity of cationic lipids arises due to inflammation and tissue damage, rapid inactivation of cationic lipids in the presence of serum
Polymer-caged nanobins	Deliver cytotoxic chemotherapeutic agents to the targeted site, thereby reducing the systemic toxicity	Scale-up of polymer-caged nanobins is in its primitive stage
Glued cell membrane camouflaged polymeric nanoparticles	Promote extensive systemic circulation, active targeting of drugs to the site of action	Large-scale production with batch- to-batch variation is a concern; quality control is another major challenge

### 2.3. Methods of Preparation of PLNs

**Figure 5:**
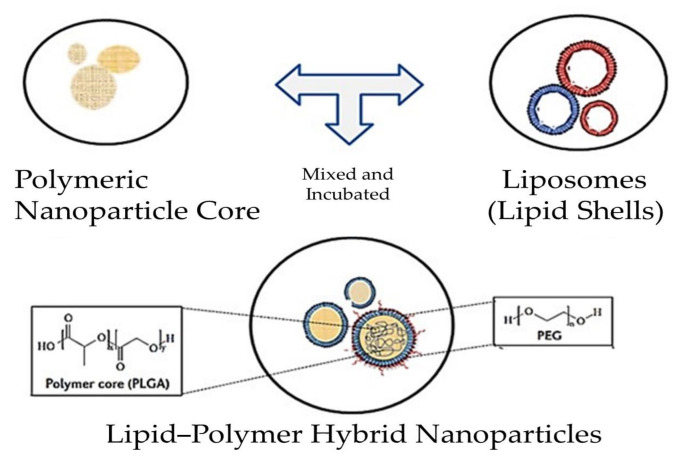
Scheme depicting the process involved in the synthesis of PLNs. The one-step approach involves the direct fusion of all the components, while in the two-step approach, components such as polymeric nanoparticle cores (PNPs) and lipid shells (liposomes) are discretely synthesized and mixed in the following steps. Finally, the PLNs are obtained.

The two approaches used to synthesize PLNs, in general, are the one- and the two-phase approaches (Figure 5). In the single-phase approach, the lipid, polymer, and drug are combined into a self-assembled nanoparticle monolayer [47]. On the other hand, in the two-phase method, the separate generation of the lipid shell and polymer core is accomplished, and both are then combined to form a bilayer of PLNs [48].

In order to synthesize a bilayer or multilayer of lipid shells for PLNs, a two-phase procedure is usually used. Polymeric kernel and lipid shell formulations are performed separately. The emulsion, nanoprecipitation, or high-pressure homogenization methods are used to create a polymer core that contains both a polymer and a drug. The sonication or extrusion of lipid-based nanoparticles or liposomes, on the other hand, may be used to build a shield around the polymeric core. Lipid membranes are formed on the surface of the polymeric nanoparticles through electrostatic interactions and the structure of the desired lipid shell–polymeric core is obtained via sonication, extrusion, high-pressure homogenization, direct hydration, or simple vortexing [47].

Since payloads and particles may be hydrophilic or hydrophobic, various synthesis methods can be used (Table 2). Conjugation is necessary to coat the polymeric core as the first step if the payload is challenging to move [49]. In brief, the polymers and payloads are dispersed in a water-insoluble chloroform-based organic solvent. An emulsion is created by dissolving the polymer solution in an aqueous solution containing the appropriate emulsifier and homogenizing it quickly. The possibility of insolubility of hydrophilic payloads in organic solvents can be reduced by preparing a polymeric core in a water-in-oil-in-water (w/o/w) double emulsion process [50]. An aqueous solution-containing payload is injected into the organic solvent encapsulated by a polymer to create a water-in-oil (w/o) emulsion. To make a w/o/w double emulsion, the resulting w/o emulsion is mixed with a second aqueous solution. The two emulsions are formed when the organic solvent is evaporated. Pre-polymerization is a general requirement for an emulsion, but high-energy homogenization causes massive polydisperse homogenate particles with a high polydispersity index. An alternative to preparing polymer core material is by pushing it over a tapered nozzle, which occurs under high pressure. If a submicron-sized droplet is spray-dried or cooled to room temperature, it permanently hardens. This technique is quick and simple to monitor, but it puts a great deal of strain on the apparatus, and the polymer particles produced are usually hundreds of nanometers in diameter.

Nanoprecipitation, on the other hand, is a popular technique for producing polymer particles smaller than 100 nanometers [51,52]. Two miscible solvents are used in this procedure, one of which is a stable polymer solvent and the other is a weak polymer solvent. After the polymer has been dissolved in the strong solvent, it is applied to the weak solvent. If the positive solvent diffuses through the weak solvent, the polymer may spontaneously precipitate out due to the complete miscibility of both phases. To mix the two solvents, dropwise addition, stirring, or sonication may be used. In addition, the modification of the polymer concentration, the volume ratio of the two solvents, and the mixing time to test the particle size and polydispersity are also carried out. A drug that corresponds to polymeric lipid hybrid nanoparticles may be made by combining polymer particles with preformed lipid or liposomes. In certain cases, lipid components are dissolved in chloroform or another organic solvent. A thin lipid film is created by evaporating the organic solvent and rehydrating the lipids with an aqueous solution. After priming the lipid films, the polymer particles may be mixed with liposomes or supplied with an aqueous solution to rehydrate them. When lipid films or liposomes are mixed with polymer centers utilizing high-pressure homogenization, high-speed vortexing, or extrusion, lipid–polymer hybrid nanoparticles are created. When the amounts of both lipids and polymer cores are handled properly, noncovalent interactions allow a lipid bilayer to develop on the surface of the polymer core. Cationic lipids form a bilayer on the carboxylic-acid-terminated PLGA polymer nucleus due to electrostatic attraction. Due to the large number of lipids in the mixture, a multilayer of lipid forms on the polymer backbone, resulting in a unique lipid cap.

The most commonly employed method to create PLNs is to render a lipid monolayer core hybridized with nanoparticles in a single stage [23]. Lipids and lipid–PEG conjugates are immiscible, so additional water-soluble solvents should be added to solubilize phospholipids in solutions. The polymer is applied to the fluid mixture of the lipid solution drop by drop. Phospholipids may be dissolved in liquid solutions with the aid of a limited volume of water-soluble solvents. To shape a polymer emulsion, the liquid polymer is carefully applied to the more viscous lipid in this expression. Lipids with a hydrophobic tail cling to the hydrophobic inner polymer nucleus, while those with a hydrophilic head group extend into the external aqueous solution. The lipid–PEG conjugate also participates in the self-assembly operation, forming a stealth cone for the nanoparticles, with its lipid moiety incorporated into the lipid monolayer and its PEG moiety facing outside of the lipid monolayer. A temperature above the lipid-phase transition temperature (The temperature required to induce a change in the lipid physical state from the ordered gel phase to the disordered liquid crystalline phase) aids in the self-assembly of lipids and lipid–PEG conjugates. A hydrophobic polymer such as PLGA should be used as hydrophobic interactions enable the self-assembled lipid monolayer to be shaped. This cost-effective, scalable, and linear lipid–polymer hybrid nanoparticle formulation technique employs a one-step self-assembly process.

The lipid–polymer hybrid NPs are made using a tweaked nanoprecipitation process combined with self-assembly. This allows NP formulation to be scalable and repeatable in clinical settings. The hydrodynamic diameter of NPs can be well controlled within the range of 50–200 nm by tuning the intrinsic parameters of the NP formulation [31].

### 2.4. Characterization of PLNs

The size, surface zeta potential, and morphology of hybrid nanoparticles decide their in vivo profiles, which are all essential physicochemical properties. Particle size is one of the most significant factors in deciding the lifetime of nanoparticles in systemic circulation and their capacity to passively accumulate in tumor tissues. For systemic drug delivery, nanoparticles with a size range of 150 nm have been shown to be highly advantageous and favorable [53]. The size distribution of nanoparticles can be determined using dynamic light scattering (DLS). This straightforward method is easy to use, and it does not require any added sample testing prior to the measurement.

However, when the particles are not perfectly spherical and have a strong polydispersity, this approach does not necessarily produce the particle’s real physical dimension. Scanning electron microscopy (SEM) or transmission electron microscopy can then be used to determine the physical measurements and structure of the particles (TEM). Valencia and coworkers [54] looked at a variety of factors that affect the size and polydispersity of lipid–polymer hybrid nanoparticles. The intense mixing of lipid and polymer solutions produces more homogeneous nanoparticles than gradual mixing. Particle size is affected by polymer agglomeration and intrinsic viscosity. Larger particles are generated by higher polymer concentrations, while smaller particles are produced by polymers with a higher intrinsic viscosity. The electrokinetic potential between the particle surface and the bulk solution is measured by the surface zeta potential, which reflects the surface electrical charges of nanoparticles and is a critical element in evaluating their stability in vitro and in vivo.

Through adding an oscillating electric field and observing the particles’ transformation when they are attracted or repulsed by the electric field, DLS may be used to measure the zeta potential of nanoparticles. Modifying the end functional groups of PEG molecules alters the surface zeta potential of lipid–polymer hybrid nanoparticles [31]. Since surface charges repel particles from colliding, higher absolute zeta potential values normally result in more stable nanoparticles in vitro. Complement activation is lowest in hybrid nanoparticles with methoxyl surface groups and highest in nanoparticles with amine surface groups according to immunocompatibility testing [54]. A sufficient surface charge must be selected to match the in vitro stability and in vivo immunocompatibility of hybrid nanoparticles. The architecture and core–shell structure of hybrid nanoparticles can be studied using electron microscopy. The nanoparticles are normally dried or fixed on a silicon wafer substrate for scanning electron microscopy (SEM) imaging, from which an exact physical scale and size distribution can be produced. A high-resolution SEM may also be used to analyze the surface morphology of hybrid nanoparticles [55], and transmission electron microscopy (TEM) is frequently used to analyze the internal core shell structure. Thevenot et al. used TEM and sodium silicotungstate for negative staining to observe lipid multilayer absorption outside a polymeric kernel. Another popular negative stain used to increase the electron density of lipids and lipid–PEG conjugates is uranyl acetate [33]. Zhang and coworkers discovered a dim ring with a thickness of less than 5 nm covering the polymer nucleus in their lipid–polymer hybrid nanoparticles prepared through a one-step self-assembly procedure [23]. Cryo-electron microscopy (CEM), which involves imaging samples at extremely low temperatures, is another valuable method for analyzing the core–shell composition of hybrid nanoparticles (usually liquid nitrogen temperature). Bershteyn and colleagues used this approach to study the impact of the quantity and composition of lipids on the lipid shell structure.

Lipid nanoparticles made up of lipids or a mixture of fatty acids, glycerides, and steroids cocooned in a single surfactant are referred to as PLNs and nanostructured lipid carriers (or in combination with a co-surfactant). The type of surfactant to use for SLN/NLC lipid nanocarrier stabilization (anionic, cationic, or non-ionic), particle size and size distribution, yield of output (YP), loading capacity (LC), and encapsulation efficiency are all determined by the lipid structure as well as the manufacturing process (EE). The lipophilicity, or ability to dissolve in a lipid matrix, of lipid nanoparticles will, of course, decide the amount of API they can store and generate. As a function of the EE, the YP and LC can be determined, which are calculated as follows:

(1) $$\;\mathrm{YP}=\frac{W_{L}}{V_{D}}\times 100$$

(2) $$\mathrm{EE}=\frac{W_{a}-{\;W}_{S}}{W_{a}}\times 100$$

(3) $$\mathrm{LC}=\frac{W_{a}-{\;W}_{S}}{W_{a}-W_{s}+{\;W}_{L}}\times 100$$

where W_L_ = the weight of lipid added in the formulation, V_D_ = the volume of the aqueous phase, Wa = the weight of API added in the formulation, and Ws = the weight of API analyzed in the supernatant (after the separation of lipid and aqueous phases by centrifugation).

As a result, EE is defined as the ratio of the entrapped API mass to total API mass, while LC is defined as the ratio of the entrapped API mass to the total lipid mass. The API’s solubility and miscibility in a melted lipid shape, the solid lipid matrix’s physicochemical structure, and the polymorphic condition of the lipid material are all factors that affect LC and EE [56].

Additionally, the entrapment efficiency is affected by the type, concentration, and crystal structure of the lipid. The drug partitioning between melted lipid and aqueous medium also affects the entrapment efficiency of nanocarriers [57]. The solubility of a drug in lipids decreases when the molten lipids are cooled; therefore, it is essential to calculate the amount of drug mixed with the lipid particles and the amount of drug solubilized in other structures within the formulation to determine the entrapment efficiency.

Based on their lipophilicity and hydrophilicity, APIs are distributed uniquely in lipid nanoparticles. Reasonably high solubility in a melted lipid is a necessary condition for achieving a high EE and LC for a specific API [57]. Due to their poor affinity for the lipid matrix, hydrophilic molecules are seldom included. Furthermore, since solubility reduces when the melt cools, API solubility could be higher in the melted lipid state than in the solid state as well as lower in the solid lipid state. Biotechnology-based APIs, on the other hand, have successfully been inserted into lipid nanoparticles, paving the way for PLNs to follow suit [58].

## 3. Optimization of Formulation Parameters by Quality by Design Approach (QbD) in the Development of PLNs

To improve lipid-based nanoformulations for lipid nanoparticles, nanostructured lipid carriers (NLCs), and nanoemulsions, a quality by design method has been used. For its processing, stability, and promotion, an efficient SLN distribution method necessitates the creation of a process flow and the definition of process control parameters. Several researchers have started to use the QbD method in the production of nano drug delivery systems in recent years to achieve better quality pharmaceutical goods by identifying and monitoring the formulation, ingredients, and manufacturing variables. QbD has been used to develop the pharmacokinetics and pharmacodynamics properties of lipid nanoparticles as well as to facilitate drug release and targeting [59].

**Figure 6:**
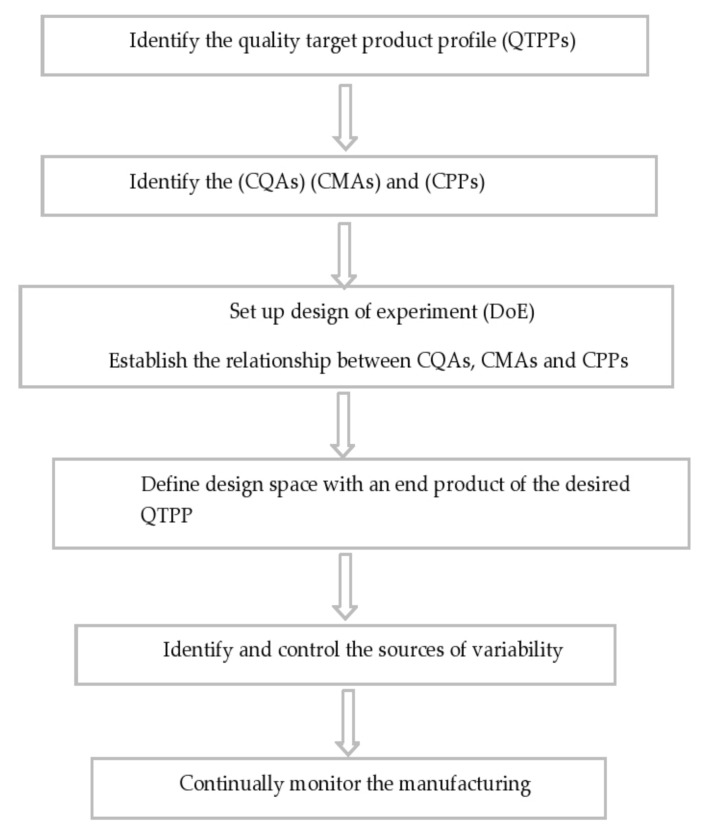
Schematic representation of steps involved in the pharmaceutical QbD implementation.

Product consistency is ensured in the existing QbD procedure by a series of steps that include raw material screening, a fixed drug product production process, and end product testing. The products are only used for processing or released onto the market if they meet all of the FDA’s or other standards’ requirements [60]. QbD is a step-by-step approach to process optimization that includes elements that, once developed, will aid in the recognition of product features and the identification of uncertainty sources [61]. Figure 6 depicts a skeletal illustration of this strategy. To summarize, the quality target product profile must first be specified and include protection and efficacy criteria. These serve as the foundation for product creation and should be replicated on a regular basis to achieve the desired results [62,63]. Following that, risk assessment is used to classify Control Process Parameters (CPPs) in a product’s production system that have a direct effect on the product’s essential quality attributes, as detailed by Control Quality Assessments (CQAs). Physical, chemical, biological, or microbiological features/specifications that are expected at a suitable limit to achieve desired product quality [63] may be included in the latter. CPPs and CQAs are identified for inclusion and investigation through the design of experiment (DoE) study using risk-assessment methods. DoE is a technique that can be used to study mechanisms and processes in order to obtain a better understanding of the primary and interaction consequences of different CPPs, as well as to forecast the intensity of these interactions [64,65].

In recent decades, nanomedicine has attracted a great deal of attention, and progress in nanosystem advancement has been at the forefront of pharmaceutical research. Drug interactions, bioavailability, and targeting are all advantages of these nanosystems that contribute to improved health conditions and fewer side effects. Industry and regulatory authorities have faced major challenges as a result of their unusual properties and variations in production methods [66,67,68]. However, controlling the efficiency and protection of nanosystems is also important. In reality, the failure of several clinical trials is due to a lack of specific guidelines for the preclinical implementation of these technologies, restricting the proposed medical use [68]. Furthermore, the large-scale transition of manufacturing processes is a barrier to industrial nanosystem processing and clinical use [66]. As a result, applying the QbD approach to the creation of nanosystems through the CPPs and CQAs as well as Process Analytical Technology (PAT) is a powerful method for identifying important production parameters and controlling variables that interfere with the quality and protection of the final goods [63]. This methodology has been used by many scholars to evaluate critical material attributes (CMAs), CPPs, CQAs, design approaches, and quantification techniques [66,67,68].

**Figure 7:**
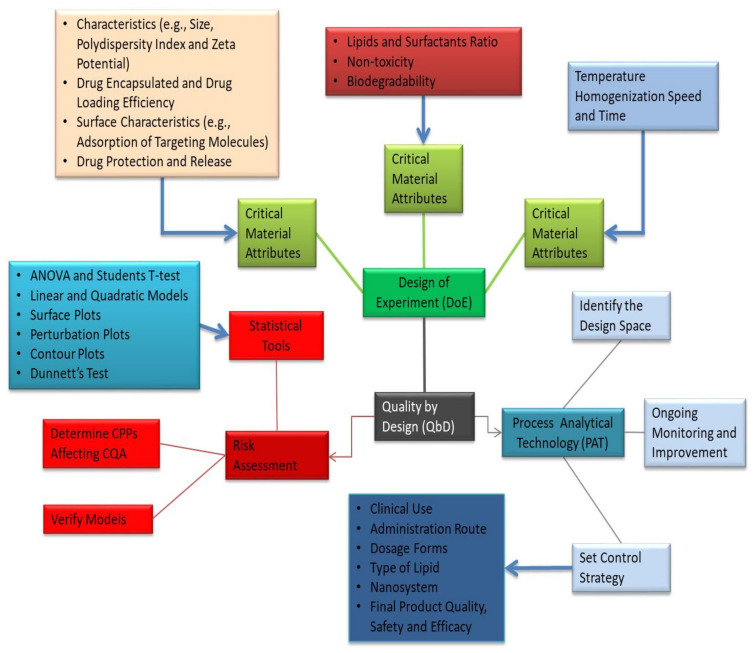
Generalized overview of QbD involved in the development of PLNs.

The quality-based design approach to the production of lipid nanosystems ensures the purity, protection, and effectiveness of the final pharmaceutical product while also allowing for effective formulation optimization (Figure 7). Variations that may occur during manufacturing are often known earlier. Raw material properties, model specifications, manufacturing methods, and scaling-up procedures are examples of these factors [69,70]. Figure 7 depicts the general QbD steps involved in the development of lipid-based nanosystems. The QbD approach is based on a prior definition of important experimental parameters. To help understand the statistical laboratory designs, such as the reaction surface methodology, Box–Behnken design, and factorial design, several experiments are used [71]. Lipid nanoparticles and PLNs are simple to make on a large scale, have a high loading potential for lipophilic molecules, provide excellent storage stability, and provide encapsulated molecules with safe and controlled release. PLNs have been used to improve drug delivery through a number of routes, including oral, cutaneous, transdermal, nasal, ocular, and parenteral [72,73,74]. QbD has been used to create lipid nanoparticle formulations that enhance pharmacokinetics and pharmacodynamic properties while still promoting successful drug release and targeting. DoE increases the final preparation’s precision by reducing the number of tests needed, which are evaluated using different mathematical models.

It is critical to optimize the formulations of the lipid nanosystems to attain reliable efficacy and safety for prescription drugs (Table 3). In the last two years, around 50 research papers have established this approach as a convenient contrivance for the consistent synthesis of nanoparticles and nanoemulsion formulations. Despite variations in quantitative models and statistical analysis, the number of tests conducted and the independent and dependent variables selected were all the same. The independent variables of lipid and emulsifier concentrations, for example, have a major impact on the dependent variables of the polydispersity index, nanoparticle/globule size, encapsulation, and zeta potential performance. In certain analyses, the impact of these independent variables on in vitro drug release development was also calculated. Manufacturing variables such as the emulsification rate and/or length, sonication amplitude and/or time, homogenization pressure and/or number of cycles, and homogenization pressure and/or number of cycles are also important when it comes to formulation optimization. QbD will be increasingly recognized as an integrative tool for the efficient production of nanosystems that complement the defined target product profile due to the use of DoE to ensure consistency within the design space as well as an appropriate risk management strategy [75,76,77].

## 4. Drug Delivery Mechanisms of PLNs

Hydrophobic drugs can be chemically bound to polymer chains in large amounts or encapsulated within the hydrophobic polymer nucleus. To prevent immune system interactions, drug delivery mechanisms should be biocompatible, hemocompatible, and immunocompatible [32]. Polymeric lipid hybrid nanoparticles (PLNs) are a reliable drug delivery vehicle for hydrophobic drugs with relatively high concentrations and sustained release levels.

**Figure 8:**
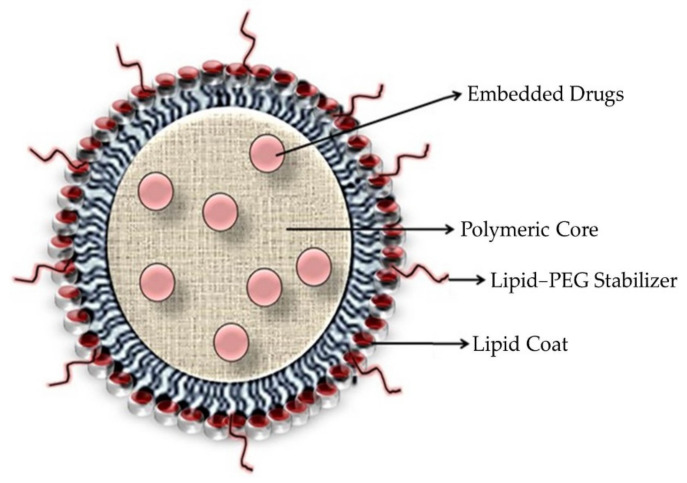
Diagrammatic representation of drug-loaded polymeric lipid hybrid nanoparticles.

The lipid coating is designed to (a) reduce water penetration into the polymeric kernel, delaying drug release and polymer degradation; and (b) prevent the free diffusion of small drug molecules out of the polymeric core, improving the drug-loading yield (Figure 8). Zhang and coworkers discovered the utilization of a shell of a lipid monolayer that amended the encapsulation as well as loading yields for an anticarcinogenic hydrophobic drug, docetaxel, as compared to polymeric nanoparticles lacking a lipid coating [23].

The critical factors that affect the drug release profiles of hybrid nanoparticles are the drug solubility, drug–polymer interaction, polymer degradation rate, and particle size. Physically encapsulated medications are released from hybrid nanoparticles using compound diffusion and polymer degradation. The release of chemically conjugated drugs is determined by the hydrolysis of the linkages between the drugs and polymer chains, as well as drug diffusion [79].

The drug molecules continuously diffuse out of the nanoparticles and into the release media. Analytical devices including mass spectrometry and high-performance liquid chromatography (HPLC) are used to isolate and measure the drugs that have been emitted or remained inside the nanoparticles at different time points [80].

In vitro assays such as neuronal absorption and cytotoxicity are often used to determine the specificity and efficacy of drug-loaded nanoparticles against target cells prior to in vivo testing. Researchers tag nanoparticles with appropriate fluorescent probes, such as fluorescence isothiocyanate (FITC), and incubate these fluorescence-tagged nanoparticles with cells to analyze the cellular absorption of nanoparticles. To visualize particle internalization and diffusion (CLSM), fluorescence microscopy, such as a confocal laser scanning microscope, may be used to view the cells after the excess particles have been removed. Once the extra particles have been removed, fluorescence microscopy, such as a confocal laser scanning microscope (CLSM), may be used to image the cells and determine whether the particles have been internalized and dispersed (CLSM).

The most common method of conducting a cellular cytotoxicity assay is to incubate drug-loaded nanoparticles with cells for a period of time. The cells are then washed and new media is inserted. After 72 h of additional culture, cell viability can be determined using appropriate assays such as the MTT and ATP assays. The drugs encapsulated inside the hybrid nanoparticles retain their cytotoxicity. Nanoparticles, in fact, greatly increase opioid toxicity as compared to free medications by delivering a bolus dosage of drugs to specific diseased cells after the particles have been internalized [81,82].

## 5. Therapeutic Applications of PLNs

PLNs are a novel drug delivery interface. By integrating two types of nanocarriers, polymeric nanoparticles and liposomes, this model architecture takes an integrative approach. These particles have a variety of properties that make them useful in the treatment of a variety of diseases, including cancers. Drug processing, gene delivery, and biomedical imaging are only a few of the medical applications for PLNs as a modern messenger system [81]. A wide variety of therapeutic agents can be encapsulated and transported using lipid–polymer hybrid nanoparticles (Table 4).

These drugs may be used alone or in combination with two or more other drugs to fill nanoparticles. During the nanoprecipitation process, hydrophobic drugs can be directly entrapped in the core of the polymer, whereas drugs that are lipophilic can be bound to the lipid coating. Drugs may be covalently bound to polymer chains to help control drug release kinetics. Chan et al. reported a promising single-drug delivery instance by inventing a “nanoburr” device to prescribe paclitaxel for the treatment of a damaged vasculature [29]. A paclitaxel-conjugated PLA center and a lecithin/DSPE-PEG shell make up the nanoburr, which is altered once more by a basement membrane-targeting peptide.

In a rat model, the sub-100 nm lipid–polymer hybrid nanoparticles accumulated in damaged vasculature and released drugs continuously for two weeks.

**Figure 9:**
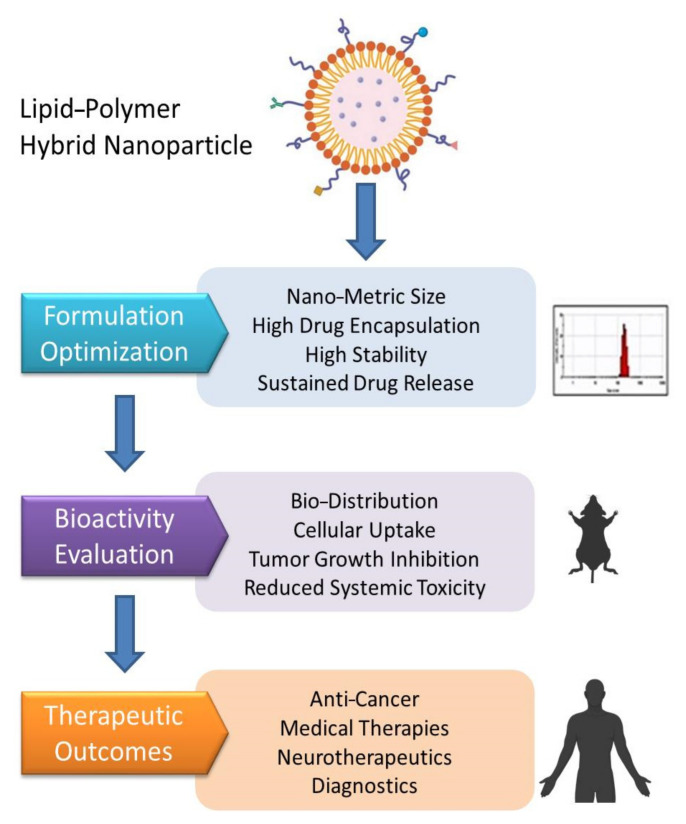
Hierarchy of the steps involved in the translation of PLNs in therapeutics.

Furthermore, the hybrid nanoparticles have shown considerable promise in the delivery of a wide range of medications (Figure 9). Wang and colleagues, for example, developed a targeted hybrid nanoparticle method for treating prostate cancer while concurrently delivering chemotherapy and radiotherapy agents [5]. During the nanoprecipitation phase, the anticancer compound called docetaxel was first encapsulated inside the polymer core, and then a radiotherapy agent called Indium-111 was applied to the particle surface and chelated to a 1,2-dimyristoyl-sn-glycero-3-phosphoethanolamine-diethylene-triamine-penta acetate (DMPE–DTPA) lipid shell. The resulting dual-drug-loaded nanoparticles had distinct drug release profiles and increased tumor cell death. Sengupta and coworkers developed a step-by-step nanocell approach to combat tumors [59]. A doxorubicin-containing PLGA polymer core and an antiangiogenic agent (combretastatin)-containing lipid multilayer shell make up the nanocell.

When compared to treatment with a single drug or a combination of the two treatments, the PLNs greatly improved tumor removal and mouse survival rates. The synergistic impact was accomplished by timing the release of the two anticancer agents: the antiangiogenesis agent was released first, triggering a rapid vascular shutdown, followed by the chemotherapy agent, which destroyed the tumor cells much more effectively. Arya et al. recently published a paper on a new approach for loading both hydrophobic and hydrophilic drugs onto lipid–polymer hybrid nanoparticles using a combinatorial drug conjugation mechanism [77]. Combination therapies aided by nanoparticles could pave the way for more effective cancer treatment in the future.

## 6. Recent Clinical Trials in PLN and Liposomal Nanocarrier-Based Therapy

Recently, because of the elevated potential to transmit medicines to therapeutic targets at reasonable levels and concentrations, nanoparticles (NPs) have attracted a great deal of attention. As demonstrated by a growing number of clinical trials, research reports, and licensed drug products, liposomes and biodegradable polymeric NPs (PNPs) have emerged as the two most popular groups of drug nanocarriers [32,83].

Liposomes, polymeric NPs, albumin NPs, and inorganic NPs are among the nanotechnology-based drugs that have started clinical trials in the last few decades, with a limited number of them now approved for clinical use [84,85]. Doxil^®^, the first successful formulation of liposomes on the market, was authorized by the regulatory authority in 1995 for ovarian cancer and AIDS-related Kaposi’s sarcoma patients. Following that, DaunoXome^®^, a daunorubicin-encapsulated medication used to treat AIDS-related Kaposi’s sarcoma, was developed by NeXstar Pharmaceuticals (Boulder, CO, USA). Depocyt^®^ (SkyePharma Inc., San Diego, CA, USA), Myocet^®^ (Elan Pharmaceuticals, Dublin, Ireland), and Mepact^®^ (SkyePharma Inc., San Diego, CA, USA) are among the liposomal medications approved for cancer care (Takeda Pharmaceuticals, IL, USA) [86]. 

Due to its biodegradability, biosafety, biocompatibility, flexibility in formulation, and functionalization, poly-lactic-co-glycolic acid (PLGA), an FDA-licensed copolymer, is one of the most widely used polymers in the design and formulation of drug delivery systems for biomedical applications, which is present at the core of polymer–lipid hybrid nanoparticles (PLNs) [87,88,89].

As evident in Table 5, the improvement in the effectiveness of PLGA-based hybrid nanoparticles in drug delivery applications is expressed in a large number of research studies published in the specialized literature as well as a large number of micro-/nanoformulations that are currently under clinical investigation or have already been licensed by the FDA [87,89].

## 7. Biopharmaceutical Aspects of PLNs

Two common methods for extending the half-life of biopharmaceutical proteins are genetic constructs or fusion approaches and conjugation with hydrophilic polymers. One method of conjugation is to use polymers including hyaluronic acid and PEG to modulate proteins. The conjugation technique has diverse benefits that encompass the ability to use a range of well-known chemistries, leading to an increased simplicity of estimation at the discovery stage using several reductionist approaches, such as a decrease in protein immunogenicity, N-hydroxy succinimide or maleimide chemistries, and a track record of several compounds. One of their disadvantages is the presence of a new molecular entity, polydispersity, as well as the possibility of polymer immunogenicity. Fusions have the advantage of being manufactured and packaged in the same manner as conventional protein therapeutics but without the need for additional downstream procedures such as encapsulation and associated costs. Furthermore, a number of products based around this approach have a proven track record. The introduction of a new molecular entity as well as the associated safety and monitoring issues, the possibility of eliciting an immune response to the modified protein, and potential formulation issues due to increased molecular complexity are all disadvantages [91].

PLGA–lipid hybrid frameworks combine the biomimetic properties of lipids with the architectural advantages of PLGA nanoparticles to form a promising carrier for a variety of difficult drugs. On the one hand, through leveraging the benefits of polymer nanoparticles, targeting the device to a particular cell receptor, and improving drug entrapment and encapsulation quality, this platform provides a viable alternative for improving customized medicine output. On the other hand, the encapsulated biological agent, which is typically correlated with lipid moieties, has a more regulated, sustained release profile. Furthermore, by limiting the number of times that a therapeutic agent is administered and by improving patient compliance, these hybrid particles reduce the systemic toxicity of the drug [87].

**Figure 10:**
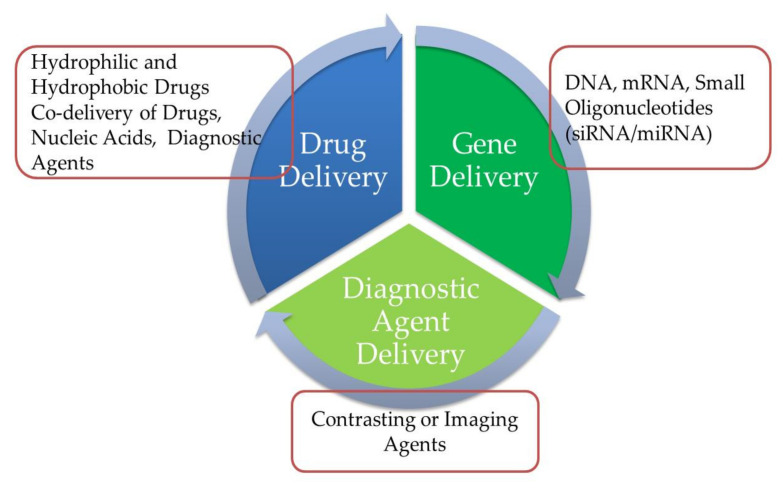
Diagram depicting the biopharmaceutical applicability of polymer–lipid hybrid nanoparticles (PLNs).

PLNs have been developed for the effective encapsulation and distribution of a broad variety of therapeutic agents, either individually or in combination. PLNs have a broad variety of applications in cancer treatment and protein-based therapeutic agent transmission, such as small interfering RNA, nucleic acid, and gene delivery, among others. Furthermore, certain medications may be delivered orally via PLNs. As seen in Figure 10, PLNs may be used to carry genes and DNA as well as vaccines and medical imaging agents [92].

If PLN-based biopharmaceuticals can be reliably delivered to the cell interior, which has several potential drug targets, they may be attractive candidates. The intracellular transmission of biopharmaceuticals is accomplished via increased membrane permeability (particularly in the case of peptides) and active transport through internalizing receptors on the cell surface, such as the asialoglycoprotein receptor on hepatocytes. Intracellular targets could include those associated with mitochondria, the nucleus, and the cytoplasm. Intracellular pathogens are another possibility [91]. Examples include Plasmodium species, mycobacteria in alveolar macrophages and associated granuloma, and Leishmania species, amastigotes in infected macrophages and various organs, including the liver and bone marrow [93].

## 8. Pharmacokinetic Properties of PLNs

The efficacy of drug therapy is dependent on the distribution of active pharmaceutical ingredients (APIs). A well-designed distribution system aims to produce an optimal degree of API at the activity site, resulting in the strongest therapeutic response and the fewest side effects possible. On the other hand, individual discrepancies in pharmacokinetic and pharmacodynamic parameters make designing dosage regimens more difficult. New innovations, such as lipid-based colloidal carriers, are being introduced to ensure proper clinical reaction. Most traditional formulations aim for immediate API availability to ensure fast and full systemic absorption. Several new API distribution frameworks have recently been introduced to enable APIs to be published at a controlled and predictable pace. Lipid-based colloidal carriers, such as nanostructured lipid carriers (NLC) and solid lipid nanoparticles (SLN), have attracted a great deal of interest in several novel delivery mechanisms. Several modified-release SLNs and NLCs optimized for different administration routes have been developed for multiple APIs based on their physicochemical and pharmacokinetic properties as well as the impact induced. For example, polymeric nanoparticles, liposomes, and regular oil-in-water (o/w) emulsions have advantages that lipid nanoparticles (SLNs and NLCs) do not have. Particles with diameters between 120 and 200 nm are seldom cleared by RES cells, preventing liver and spleen filtration [92], and various release profiles may be obtained since the API is inserted into the lipid matrix [94,95,96]. Particles with longer circulation times and hence greater ability to target the site of interest should be 120–200 nm in diameter and have a hydrophilic surface (external hydrophilic polymer corona) in order to reduce the clearance by macrophages.

One of the most significant disadvantages of drug delivery nanoparticles is the rapid clearance of carrier from the bloodstream by the reticulo endothelial system (RES). Following intravenous administration, the carriers first come into contact with plasma/serum proteins (opsonins) before they reach the target cells. The opsonins adsorb onto the surface of the carriers (opsonization) and render the particles recognizable and more palatable to RES, enabling the particles to be extracted from the bloodstream (MPS) [97]. According to the findings of [98,99], particle size, surface charge, PEG alteration, and targeting ability are all important factors in deciding the in vivo behavior of drug delivery nanoparticles. PEGylated nanoparticles with a size range of 100 nm and a slightly negative surface charge are thought to be able to stay in the systemic circulation systems for hours and extravagate preferentially into tumor tissues through passive diffusion and active targeting properties [52]. Due to their unique core shell structure and flexibility in controlling scale, surface charge, and surface functionalization, polymer lipid hybrid nanoparticles carry a great deal of promise for achieving favorable in vivo pharmacokinetic properties. Sengupta and colleagues demonstrated that their polymer–lipid hybrid nanoparticles, or nanocells, have outstanding pharmacokinetics and therapeutic efficacy in the treatment of melanoma and Lewis lung carcinoma, respectively [99]. Since hybrid nanoparticles are a relatively new drug delivery technique, there is a limited amount of in vivo data, while rigorous in vivo trials are currently being conducted in various research laboratories. Targeted distribution is another important aspect of nanoparticle drug delivery technology. A common method of effectively directing nanoparticles to action sites is to conjugate ligands specific to cells or tissues onto the surface of the particles. Although polymer–lipid hybrid nanoparticles (PLNs) have shown efficacy in cell culture studies, further in vivo study is required to determine their tissue targeting potential [100].

Polymer–lipid hybrid nanoparticle drugs are able to maintain local bioavailability, according to Zhang et al. In comparison to a free drug, they exhibit greater tumor cell apoptosis and non-detectable systematic and organ toxicity. The superior pharmacokinetics, biodistribution, and effectiveness of PLNs pave the way for better nanomedicine design in the future, which can be expected to hit the pinnacle of performance [100].

## 9. Limitation of PLNs

The development of innovative methods for drug delivery, such as polymeric lipid hybrid nanoparticles, is crucial for efficient drug delivery, but the ability to reliably monitor and simulate the effects of these models is crucial for clinical translation performance. Besides that, it may also be crucial to filter and identify PLNs with the highest quality for a specific use, necessitating the consistent processing of PLNs with specific charges, sizes, and ligand densities [101].

## 10. Conclusions and Future Perspectives

Polymeric lipid hybrid nanoparticles (PLNs) were first developed in the early 2000s to solve the issue of inserting ionic drugs into a hydrophobic stable lipid phase that are water-soluble in order to ensure sufficient loading and continuous release of active molecules [102]. Many PLN formulations have been developed since then to encapsulate a single anticancer drug or to co-encapsulate a drug along with a chemosensitizer [103]. In recent years, PLNs for gene therapy and immunotherapy, such as small interfering RNAs in combination with an anticancer drug, have attracted a great deal of attention [104]. In cancer cells, PLN formulations have been shown to resolve efflux transporter-mediated multidrug resistance (MDR) and improve antitumor potency while reducing systemic toxicity from anticancer drugs [104,105].

PLNs have been recognized as a seamless and efficient medication distribution mechanism in contrast to other types of drug delivery systems because they are simpler to prepare and have superior stability. PLNs may be used for a variety of applications, including anticancer therapy and bioimaging, due to their small size. Furthermore, PLNs encase drug molecules such as proteins, DNA, and peptides in a lipid–PEG coating, which protects them from degradation. Future research will explore the possibility of converting PLNs to dry powder while maintaining their physical properties and durability. A variety of PLNs have been approved in clinical trials, and some are in the early stages of production [106].

The ultimate aim of research into PLN-mediated drug delivery is to create a clinically successful and secure treatment. In cancer treatment, where toxicity is often related to clinical efficacy, developing PLN formulations with improved potency and less harmful side effects is critical. Over the last 15 years, the area of PLN-mediated drug delivery has gained traction to accomplish this aim. Many of the PLNs on this list are made up of lipids, polymers, and surfactants that are either only used in licensed pharmaceutical drugs or are usually considered harmless.

Integrating polymer and lipid effects in PLN opens up a number of possibilities for developing PLNs to meet a range of drug delivery needs, particularly for combination therapies involving multiple agents with different properties. The PLN platform’s specific advantages and great potential have already been demonstrated in field testing, necessitating further progress.

When developing PLN formulations, simple pharmacokinetic properties of nanoparticle drug carriers such as their drug-loading capacity, particle size, loading efficiency, surface charge, stability, and spatiotemporal drug release should all be considered [107]. Physicochemical compatibility and affinity are needed for efficiently loading medication into PLNs and precisely controlling drug release kinetics. As a consequence, a thorough understanding of material properties in relation to physicochemical processes is needed for effective PLN design.

The demand for nanocarrier-based drug delivery systems for oral therapeutic delivery has gradually risen over the last few decades. Nanocarriers have two main advantages: high uptake and drug molecule stabilization in the GI tract. PLNs are more beneficial than other nanocarriers produced for oral distribution. They have a complicated form, but they are also easy to prepare using convenient methods. They are widely used in all fields of medicine, including cancer treatment, gene transfer, nucleic acids, siRNA, etc. They can also be used to encapsulate hydrophilic and hydrophobic medicines for oral administration. The PLNs have a long and regulated release time as well as outstanding in vivo properties. These carriers present a large number of possible options for delivering therapeutics through the oral route in the future.

## Figures and Tables

**Table 2:** Comparison of formulation techniques used to prepare the polymeric lipid hybrid nanoparticles [46].

Techniques Used	Advantages	Drawbacks
Single-step method	Less time consuming, a preferred technique for designing core–shell-type hollow lipid–polymer nanoparticles	The use of organic solvents limits this method to prepare nanoparticles
Two-step method	Sonication and extrusion enable small uniform particle size with a low polydispersity index (PDI). Controlled drug release due to the polymeric core and lipid shell The theoretical amount of lipid required to uniformly coat the polymeric core can be calculated from the core and phospholipid properties	Reduction in the encapsulation efficiency of water-soluble drug during the incubation process. Less efficient technique
Modified nanoprecipitation method	By optimizing the polymer to lipid ratio, particle size and polydispersibility can be controlled	The use of organic solvents is a major drawback, and it is time consuming

**Table 3:** Optimization of PLN formulation parameters using the QbD approach.

Mathematical Model (Design of Experiment (DoE))		Statistical Analysis	Independent Variables (Critical Material Attributes (CMAs) and Critical Process Parameters (CPPs))	Dependent Variables (Critical Quality Attributes (CQAs))
Box–Behnken Method [78]	3^3^ factorial Box–Behnken design	ANOVA and student’s *t*-test Bonferroni’s post hoc test	Concentration of drugs	Particle size
	3^2^ Box–Behnken design	ANOVA and Dunnett’s test	Concentration of lipids	Particle size
Plackett–Burman design		Pareto chart	Concentration of emulsifier	Polydispersity index
Central composite design		Linear model	Lipid–drug ratio Concentration of emulsifier	Zeta potential Ex vivo drug permeation
Factorial design [66]	3^2^ factorial design	Linear and Quadratic models	Concentration of lipid Concentration of emulsifier	Polydispersity index
	2^3^ full factorial design	Surface plot (fitted response)	Lipid–drug ratio Concentration of emulsifier	In vitro drug release
	2^4^ full factorial design	Surface and contour plot (fitted response)	Concentration of lipid Concentration of emulsifier	Percentage yield
Orthogonal design		Contour and perturbation plots	Homogenization speed	Encapsulation efficiency
Taguchi		Two-factor interaction	Homogenization speed, pressure, and time	Zeta potential
Resolution IV experimental design [78]		Counter and surface plot	Homogenization speed, pressure, and time Sonication amplitude/ time	Zeta potential Encapsulation efficiency

**Table 4:** Therapeutic applications of PLNs [29,83].

Polymers Used	Lipids Used	Targeting Ligand/Combinatorial Drug Delivery	Drugs	Applications
PLGA	Lecithin/PEG_2000_/DSPE–PEG_2000_-FA	Folate	Doxorubicin Hydrochloride	Enhanced and selective targeting to folate receptor-positive cancer cells in vitro
PLGA	DLPC/DSPE–PEG_2000_/DSPE–PEG_2000_-FA	Folate	Docetaxel	Maximal accumulation and penetration into the tumour cells overexpressing folate receptors
PCL–PEG–PCL	PEG/DSPE–PEG_2000_/DSPE–PEG_2000_-FA	Folate	Paclitaxel	Intratumoral delivery of paclitaxel-loaded folate-targeted hybrid nanoparticles showed lower toxicity and greater therapeutic efficacy
PLGA	DSPE–PEG_2000_/lecithin/DSPE–PEG_2000_-FA	Folate	Cisplatin/Indocyanine green	Combined chemo-photothermal therapy. Induced apoptotic cell death and inhibited the tumor recurrence
PLGA	Lecithin/DSPE–PEG-COOH/ DSPE–PEG_2000_-FA	Folate	Doxorubicin	Enhanced cellular uptake and cytotoxicity in folate overexpressing human oral cavity squamous carcinoma cells and showed greater tumour accumulation and appreciable antitumor efficacy
PLA	SPC/DPPE/DSPE–PEG-COOH/ DSPE–PEG_2000_-FA	Folate	Mitomycin C	Improved pharmacokinetic profile (compared with free drug) by extending circulation time and showed better in vitro and in vivo therapeutic efficiency
PLGA	EPC/DSPE–PEG/DSPE/H2N-PEG2K-OH	RGD	10-Hydroxy camptothecin	Enhanced cytotoxicity profile of hydroxy camptothecin
mPEG–PLGA	Lecithin/cholesterol/Chol-PEG-RGD	RGD	Curcumin	Enhanced cytotoxicity of curcumin in vitro and prolonged survival in a subcutaneous B16 murine tumour model
PLGA–COOH	Lecithin/DSPE–PEG_2000_-Maleimide	RGD	Isoliquiritigenin	Demonstrated better cytotoxicity and apoptotic cell death of different types of breast cancer cells, prolonged in vivo circulation and exhibited higher tumor growth inhibition efficacy in 4T1-bearing breast tumor murine models
PLGA	Lecithin/DSPE–PEG_2000_-OMe/ DSPE–PEG_2000_-RGD	RGD	Docetaxel	The median survival times for the rats treated with RGD-functionalized docetaxel-loaded NPs were prolonged by 57 days after a series of experiments
PLGA	Lecithin/DSPE-PEG	Doxorubicin	Indocyanine green	Apoptotic cell death and inhibited tumor recurrence
PLGA	PEG-DSPE/phosphatidyl choline/cholesterol	Doxorubicin	Combretastatin	The therapeutic efficacies are validated in the murine model of melanoma and Lewis lung carcinoma
PLGA	Lecithin/DSPE-PEG-COOH	Paclitaxel	Gemcitabine hydrochloride	Showed enhanced cytotoxicity over their single counterparts
Poly-L-arginine/PLA/PEI	DSPC/cholesterol/POPG	siRNA	Doxorubicin	siRNA that downregulates a drug-resistant pathway and doxorubicin to treat triple-negative breast cancer in an MDA-MB-468 xenograft model

Abbreviations: DSPE–PEG–FA—folate-modified distearoylphosphatidyl ethanolamine–polyethylene glycol, PCL—poly (έ-caprolactone), EPC—O-alky phosphatidyl choline, RGD—arginylglycyl aspartic acid, PLA—poly lactic acid, PEI—polyethyleneimine, PLGA—poly-lactic-co-glycolic acid, DLPC—dialuroylphosphatidyl choline, POPG—palmitoyloeoylphosphatidyl glycerol.

**Table 5:** Status of clinical trials of PLGA-based nanoparticles [27,90].

Name	Drug	Architecture of PLNs	Investigated Applications	Company	Status	Year
Lupron Depot^®^	Leuprolide	Sterile lyophilized microspheres in which the leuprolide is incorporated in a biodegradable copolymer of lactic and glycolic acids	Prostate cancer, endometriosis, central precocious puberty	Abbot Laboratories, Takeda	Approved	1989
Sandostatin Lar^®^	Octreotide acetate	Long-acting repeatable depot formulation consisting of biodegradable glucose star polymer, D,L-lactic acid, and glycolic acid copolymer	Acromegaly	Novartis	Approved	1998
Trelstar^®^	Triptorelin pamoate	Sterile lyophilized, biodegradable microgranule formulation containing triptorelin pamoate, PLGA, mannitol, carboxy methyl cellulose, and polysorbate 80	Prostate cancer	Allergen	Approved	1998
Arestin^®^	Minocycline HCl	Subgingival sustained release of microspheres containing bioresorbable polymer poly (glycolide-co-dl-lactide)	As an adjunct in adult periodontitis	Bausch Health U.S.	Approved	2001
Eligard^®^	Leuprolide acetate	Sterile polymeric matrix formulation consisting of a biodegradable poly(D,L-lactide-co-glycolide) (PLGH or PLG) polymer forms a solid drug delivery depot	Prostate cancer	Tolmar	Approved	2002
Risperdal Consta^®^	Risperidone	Extended release microspheres formulation of risperidone encapsulated in polyglactin	Schizophrenia and bipolar I disorder	Janssen Pharmaceuticals Inc.	Approved	2003
Vivitrol^®^	Naltrexone	Extended release injectable suspension containing poly(lactide-co-glycolide)	Opioid antagonist	Alkermes Inc.	Approved	2006
Ozurdex^®^	Dexamethasone	Intravitreal implant contains micronized dexamethasone in a biodegradable polymer matrix	Corticosteroid	Allergan Inc.	Approved	2009
Bydureon^®^	Exenatide synthetic	Extended release injectable containing poly(D,L-lactide-co-glycolide) polymer along with sucrose	Type II diabetes	AstraZeneca AB	Approved	2012
Signifor Lar^®^	Pasireotide pamoate	It consists of pasireotide pamoate uniformly distributed within microspheres containing biodegradable copolymers of poly (D,L-lactide-co-glycolide) acids	Acromegaly	Novartis	Approved	2014
Zilretta^®^	Triamcinolone	Suspension of microspheres consisting of small crystals of triamcinolone acetonide, embedded in a poly-lactic-co-glycolic acid co-polymer matrix	Osteoarthritis and other corticosteroid therapy	Flexion therapeutics Inc	Approved	2017
Bydureon Bcise^®^	Exenatide	Extended release sterile microsphere suspension in an oil-based vehicle of medium chain triglycerides (MCT)	Type II diabetes	AstraZeneca AB	Approved	2017
Triptodur Kit^®^	Triptorelin pamoate	Sterile, lyophilized, biodegradable microgranule formulation, comprised of triptorelin pamoate, poly-D,L*-*lactide-co-glycolide, mannitol, carboxymethylcellulose sodium, and polysorbate 80	Central precocious puberty	Arbor	Approved	2017
Sublocade^®^	Buprenorphine	Extended release injection in which buprenorphine is dissolved in the ATRIGEL^®^ delivery system The ATRIGEL^®^ delivery system consists of biodegradable poly(D,L-lactide-co-glycolide) polymer and a biocompatible solvent, N-methyl-2-pyrrolidone	Moderate to severe addiction to opioid drugs	Indivior	Approved	2017

## Data Availability

Not applicable.
